# Foreign

**Published:** 1841-03-20

**Authors:** 


### FOREIGN.

We published in a former number of the Examiner the statistical tables of Drs. Norris and Hayward, exhibiting the results of Great Amputations, during a series of years in the Pennsylvania Hospital, and in the Massachusetts General Hospital. From these, many learned with surprise, that even under circumstances so favourable to success, the average mortality was a fraction more than 1 in 4. We are glad to perceive that these statements have received a degree of attention abroad, commensurate with their importance, and have elicited additional facts, which all confirm the position of Drs. Norris and Hayward, that the dangers of large amputations have hitherto been grossly underrated. We expressed at the time our regret, that these tables were so limited in their object. They were intended to display merely the average mortality; they clearly exhibited the dangers of large amputations, while they offered no indication by which these dangers could be lessened or avoided.

In the admirable paper by Dr. Lawrie, which we quote below, we are glad to see that the author coincides with our views of the necessity of rendering such statistics more comprehensive, if we wish to give them their full practical value. The additional facts deducible from his tables, prove him to be right. Thus a comparison of the proportionate number of deaths after amputation of the leg, in reference to the points where the bones were sawed, has already served to confirm the opinion we entertained, that amputations near the ankle were less dangerous than those higher up, and consequently that the very general practice of operating immediately below the knee, should be abandoned when there is a choice, and as much of the leg be preserved as possible.

But by far the most important addition to the statistics hitherto published, is to be found in the last table of Dr. Lawrie, which shows "*the causes of death* in 73 amputations, the limbs amputated, and the number of days each case survived."

Cases of amputation are so numerous, and can be so rigidly classified, that the results of their analysis may be regarded as more exact than could be derived from any other source. The causes productive of death are not to any great degree peculiar to amputations, but are common to them and other great surgical operations. The present spirit of inquiry in regard to this class is therefore calculated, if properly directed, to throw the clearest light upon the dangers of operations in general, and possibly to open a road for their future avoidance. It is with this belief that we lay before our readers the paper of Dr. Lawrie.

*On the Results of Amputations.* By J. A. LAWRIE, M. D.

The frequency of its performance, the mutilation which it causes, its severity, and its immediate and ultimate dangers, combine in making the operation of amputation one of the most important subjects which can occupy the attention of the practical and operative surgeon: it is peculiarly so to the hospital surgeon in such a city as Glasgow. In private practice, in the better ranks of society, it is an operation of comparatively rare performance; whereas in hospitals it exceeds many fold all the other capital operations combined. In this city and neighbourhood, the character of the climate strongly predisposing to diseases of the joints; the number of our public works; the extent and variety of machinery employed in our manufactories; the amount of our shipping, and number of steam vessels; our coal mines and stone quarries; the great increase of building, and the certainty that ere long Glasgow will be the centre from which numerous rail-ways will radiate, give to the operation of amputation, by greatly increasing its frequency, an interest exceeding perhaps all others performed in our infirmary.

I need not inform the members of this section that this subject has attracted much attention, and that of late years many parts of it have been so much improved as to leave little farther to be desired. The subject of this essay, "The Results of Amputations," has not, however, met with that consideration to which its importance appears to entitle it. Although success may not in every instance be a fair test of the propriety of treatment adopted in individual cases; in the aggregate, it is the best—indeed, the only true—criterion by which to judge of operative surgery. For many years the operation of lithotomy, and the removal of cancerous disease have been tested in this manner, and the result has been that the confidence of the profession has increased in the one, while the other, except as a palliative, has been almost entirely abandoned. I am surprised that the same severity of investigation has not been applied to amputations. Mr. Samuel Cooper, in the last edition of his Dictionary, while he devotes several columns of his closely printed pages to the history of amputations, says scarcely a word of their results.

Our best and most recent works on operative surgery are open to the same objection; for while the different methods which ingenuity has suggested for the removal of particular parts are minutely detailed, hardly one word is said of the results of their operations, either in the aggregate, or as contrasted with each other. Our military surgeons for many years have been more attentive to this subject than their brethren in civil practice; but until the end of the peninsular war their opinions were by no means in accordance regarding it. Belguer, Surgeon-General to the Prussian army, in a treatise published in 1762, condemns amputation as an operation hardly justifiable under any circumstances. He carried his opposition so far, that "he suffered no amputation to be performed in the Prussian army." He states his success as follows:—I had at one time during the war, in a military hospital, 6618 wounded patients; of them 5557 were perfectly cured, 195 were able to do duty in person or to work at any trade, 213 remained incapable of any labour, civil or military, and 663 died. The 195 and 213 invalids were of the number of those who had their bones broken and shattered, or, in other words, whose wounds were complicated and dangerous. Of the 663 who died, 408 only, died from shattered bones, which number, 408, is equal to that of those who were cured without amputation, although their wounds had been of the same kind." One half therefore of his severe wounds of the extremities died, the other lived without operation. "If," continues Belguer, "after making these calculations, we compare them with the prodigious number of married men who, at the beginning of the war, had their limbs taken off on account of dangerous wounds, of whom scarcely one or two escaped with their lives, we may safely conclude that much the greater part of these 408 men cured and sent to the invalids, would have died if amputation had been performed."

The result of Belguer's experience would thus seem to be, that of 6618 wounded, 916 were cases requiring amputation, of whom 408 died, 213 were incapable of any labour, and 195 were able to work at any trade.

These opinions do not seem to have influenced the practice of British military surgeons, who never seem to have doubted the propriety of amputations in certain severe accidents. With them the preference to be given to primary or secondary amputations after gun-shot injuries, formed the principal ground for difference of opinion. Wiseman and Manby were in favour of immediate amputation, while John Hunter was an advocate for the secondary. Towards the close of the peninsular war, British military surgeons were almost unanimously in favour of the former. Mr. Guthrie, in his work on Gun-shot Wounds and Amputations, gives the following tables:—

I. *Amputations performed at hospital stations.—Secondary.*

| | *No. of Amputations.* | *Died.* | *Discharged Cured.* | *Under Treatment.* |
|---|---|---|---|---|
| Amputations of upper extremity - | 296 | 116 | 105 | 75 |
| Amputations of lower extremity - | 255 | 149 | 65 | 41 |
| Total, | 551 | 265 | 170 | 116 |

II. *Operations performed on the field of battle.—Primary.*

| | *Number of Cases.* | *Died.* | *Discharged Cured.* | *Under Treatment.* |
|---|---|---|---|---|
| Amputations of upper extremity - | 163 | 5 | 64 | 94 |
| Amputations of lower extremity - | 128 | 19 | 43 | 66 |
| Total, | 291 | 24 | 107 | 160 |

From these tables it appears that of 551 cases of secondary amputations, 265, or nearly one half, died. Of 291 cases of primary amputations, 24, or nearly one in twelve, died. Farther, the comparative loss in secondary or delayed operations, and primary or immediate, is—

| | *Secondary.* | | *Primary.* |
|---|---|---|---|
| Upper extremities - - - - | 12 | to | 1 |
| Lower extremities - - - - | 3 | to | 1 |

These tables appear quite conclusive as to the propriety of primary amputation in military practice. In other respects, however, they are not so satisfactory as could be wished. The cases marked "under treatment," in both tables, having passed the periods of danger, are considered as recovered. In civil hospital practice, I would not consider a patient recovered until he had left the hospital; and as the cases under treatment amount to 276, they cannot be looked upon as accurate data for estimating the results of amputations.

In the *Medical Gazette* for June 1838, there is a paper by Mr. Phillips, entitled "Observations arising out of the Results of Amputations in different Countries." He says (p. 459,) the amputations included in this inquiry are those of the arm and forearm, leg and thigh. The whole of them have been performed in the last four years in civil hospitals, and in the private practice of hospital surgeons. The details are—

| | *Cases.* | *Deaths.* | *Per Cent.* |
|---|---|---|---|
| France - - - - - - | 203 | 47 | $23\frac{31}{203}$ |
| Germany - - - - - | 109 | 26 | $23\frac{93}{109}$ |
| America - - - - - | 95 | 24 | $25\frac{5}{19}$ |
| Great Britain - - | 233 | 53 | $22\frac{174}{233}$ |
| Total | 640 | 150 | $23\frac{1}{7}$ |

By these returns it appears, that the average number of deaths is nearly 23½ per cent. The remainder of Mr. Phillips's paper is devoted to the question of immediate or secondary union of stumps after amputation for disease, the result of which is, that "attendant on the practice of immediate union is a mortality amounting to 25 per cent."

It must be obvious that the experience of any one of even our best employed surgeons must be inadequate to determine a question of such extent as the results of amputations. Experience on a large scale, and extended over a considerable period can alone be relied on. It occurred to me, that the records of an infirmary, carefully examined, might in a great measure supply the deficiency, and that details more minute than any I have yet met with, might be of use to our hospital surgeons. Assisted by my young friends, Dr. M'Lean and Mr. Parish, I have carefully examined all our infirmary surgical records which I could procure, from the commencement of the hospital to the beginning of 1839, and I have now the pleasure to lay the results before this section. They are neither so extensive, nor, in many instances, so minute as I could wish. Many of the journals have been lost, and others are very carelessly kept; some, on the other hand, are so admirably accurate, that had all the surgical and medical records of any infirmary, from the year 1795 to the present day, been as carefully kept, they would have presented a mass of information capable of determining almost any point in the professional statistics of hospital practice. We are not yet too late, and I would take the liberty of impressing on the members of this section, connected with hospitals, the great value of accurate records. The present state and history of every case ought to be entered in a journal kept for the purpose, and belonging to the hospital, which ought to be premised by a statement of the patient's name, age, constitution, occupation, and residence. (This last is of importance, as enabling us to trace our patients after they leave the hospital, and to ascertain the ultimate result of our treatment.) The date of each operation should be accurately noted, and the true results candidly given. It would also be a convenience if the indices were properly filled up with a column for "operations."

The abstract from which these remarks and tables are drawn consists of 276 cases of amputation of the shoulder, arm and forearm, hip, thigh, leg, and foot, as they occur in the journals, from 1794 to the end of 1838.

Of these 276 there were—

| | | |
|---|---|---|
| Cured | 176 | 63.7 per cent. |
| Died | 100 | 36.3 " |

Deaths to recoveries as 1 to 2.75

a mortality, I fear, greater than those are prepared for, who have not been in the habit of turning their attention to this subject.

Of the 276 there were—

| | | *Cured.* | *Died.* | *Deaths to Recoveries.* |
|---|---|---|---|---|
| Males | 216 | 130 | 86 | 1 to 1.6 |
| Females | 60 | 46 | 14 | 1 to 3.3 |

By which it appears that the males are to the females as $3\frac{1}{2}$ to 1, and that the proportionate mortality among the males is to that among the females as 1.6 to 3.3. The greater mortality among the males is in part, but not altogether, accounted for by the greater number of primary amputations among them, as will appear in the sequel.

Of 133 cases in which "the number of days in hospital after amputation" is noted, 79 were cured, 54 died. Of the cured, the average number of days under treatment after operation is 42, the least being 8, the greatest 128. Of the deaths, the average is 13 days, the least three hours, the greatest 199 days.

I have divided the cases of amputation into two general classes: 1. Amputation for disease: 2. Amputations for injuries. The second I have subdivided into primary and secondary. By primary amputations, I mean amputations for injuries performed as soon after the accident as circumstances will permit —almost always within the first 24 hours, and without any attempt made to save the limb. By secondary, those cases in which attempts have been made, and failed, to preserve the limb, and amputation been performed in consequence of the failure. Each of them I have subdivided into amputations at the shoulder, arm and forearm, hip, thigh, and leg.

I. *Amputations for disease.*—Of the 276 cases, 153 (more than one-half) were for disease.

| | | |
|---|---|---|
| Cured | 118 | 77.1 per cent. |
| Died | 35 | 22.9 " |

The following table shows the detailed results:—

| | *No. of each.* | *Cured.* | *Died.* | *Deaths to recoveries as* |
|---|---|---|---|---|
| Shoulder - - | 2 | 1 | 1 | 1 to 1 |
| Arm - - - - | 17 | 14 | 3 | $1\frac{1}{2}$ to 7 |
| Foream - - - | 4 | 4 | 0 | No death. |
| Thigh - - - | 92 | 73 | 19 | 1 to $3\frac{3}{4}$ nearly |
| Leg - - - - | 35 | 23 | 12 | 1 to 2 fully |
| Foot (partial) | 3 | 3 | 0 | No death. |
| Total | 153 | 118 | 35 | |

The diseases with their results were as follows:—

| | Of which there were— | Males | | Females | | Total | Total | Deaths to Recoveries. |
|---|---|---|---|---|---|---|---|---|
| | | Cured. | Died. | Cured. | Died. | Cured. | Died. | |
| Diseased joints | 98 | 60 | 11 | 23 | 4 | 83 | 15 | 1 to 6 1-5th |
| Necrosis | 12 | 7 | 1 | 2 | 2 | 9 | 3 | 1 to 3 |
| Caries | 21 | 13 | 4 | 3 | 1 | 16 | 5 | 1 to 3 1-5th |
| Tumors | 12 | 6 | 2 | 3 | 1 | 9 | 3 | 1 to 3 |
| Gangrene | 5 | 1 | 1 | 2 | 1 | 3 | 2 | 2 to 3 |
| Ulcers | 5 | 1 | 4 | 0 | 0 | 1 | 4 | 4 to 1 |

In this table of the more common amputations, that below the knee is the least favourable. This may be owing to the unfavouarble situation at which the operation is performed. In a great majority of instances, even for disease of the foot and ankle, amputation of the leg is performed below the knee, to enable the patient to rest on the bent knee, in using the common "wooden pin." But by a very simple, and by no means expensive apparatus, the patient can walk on the "wooden pin" with his knee straight, and a long stump, instead of being an inconvenience, is a great advantage. The above tables, by pointing out the danger of the common method of operating, should induce us never to remove more of the limb than will insure that the parts which form the stump are sound, and in all cases except those of necessity, to abandon the operation "below the knee."

It struck me as remarkable, that of the first 30 amputations in the list "for disease," viz., from the year 1794 to 1810 inclusive, 29 recovered, and one died; whereas, of the last 30, 22 recovered, and 8 died. Thinking this might be accidental, I took the central numbers

(from 142 to 209, inclusive,) and the proportion was 19 cures, and 11 deaths. It is not altogether easy to account for this very remarkable discrepancy. It is obviously by no means complimentary to our modern improvements in operating and dressing. Of the first 30, in which the cures are to the deaths as 29 to 1, it is probable that all the operations were performed by the double circular method, that all the stumps were dressed before the patient left the table, that the dressings were removed by rule, with little regard to the appearance of the stump, and that in all the tourniquet was used. Of the last 30, of which 22 recovered, and 8 died, 28 were single or double flap operations, of which 21 recovered, and 7 died; 2 were double circular, of which one was cured and one died.

Of the *supposed* recent improvements, there is one which I may be excused for noticing in this place,—I mean delayed dressing. This after what I thought a fair trial, I have almost entirely abandoned. Its advocates support it mainly on the plea of avoiding secondary hæmorrhage. In my own practice I do not recollect ever to have taken down a stump for this cause, and I believe a little care in tying bleeding vessels on the operating table would prevent it. My objection to the plan of not dressing the stump till six or eight hours after the operation, is the additional suffering that it causes our patients. I have uniformly found my patients complain fully more, and suffer nearly as great a shock from the delayed dressing as from the operation itself. As for preventing hæmorrhage, the support of the dressings frequently has this effect; and as for the pain of removing the dressings in the few cases in which hæmorrhage does occur, I believe it to be small in amount in comparison with that which must be occasioned in every case by not dressing on the table.

It is probable that, previously to the publication of Sir B. Brodie's work on Diseases of the Joints, diseased articulations, especially of the knee, classified under the comprehensive appellation "white swelling," were reckoned incurable, and removed at a much earlier period than they now are. Of this I am convinced from a perusal of the cases, as recorded in the journals of the Infirmary. Amputation seems to have been performed for disease, the very proposal of removing which by operation a modern consultation would scout. To prove this I constructed the two following tables. I took the 71 first cases of diseased joints as they occur in the journals, from 1794, downwards, and 82 in the journals since 1833, without selection. The numbers 71 and 82 are accidental.

*Tables showing the results of diseased joints, treated in the earlier and later periods of the hospitals*

| | *Amputated.* | | *Not Amputated.* | | | No of |
|---|---|---|---|---|---|---|
| *Early period.* | Cured. | Died. | Cured. | Relieved. | Died. | Cases. |
| Elbow - - - | 5 | 0 | 1 | 6 | 0 | 12 |
| Wrist - - - - | 1 | 0 | 1 | 2 | 0 | 4 |
| Knee - - - - | 16 | 3 | 9 | 15 | 1 | 44 |
| Ankle - - - - | 5 | 0 | 2 | 4 | 0 | 11 |
| Totals | 27 | 3 | 13 | 27 | 1 | 71 |
| | 30 | | 41 | | | |
| *Late period.* | | | | | | |
| Elbow - - - | 3 | 0 | 2 | 6 | 0 | 11 |
| Wrist - - - - | 2 | 0 | 3 | 0 | 0 | 5 |
| Knee - - - - | 8 | 3 | 24 | 20 | 2 | 57 |
| Ankle - - - | 4 | 2 | 2 | 1 | 0 | 9 |
| Totals | 17 | 5 | 31 | 27 | 2 | 82 |
| | 22 | | 41 | | | |

From these tables it appears, of 71 cases of diseased joints in the early days of the hospital, 30, or 1 in 2.36, suffered amputations; while of 82 in the later period, 22, or 1 in 3.73, only were operated on. The difference is still more striking if we take the knee joint. Of 44 in the early period, 19, or 1 in 2.30, were amputated; while, at the later period, of 57, 11, or 1 in 5.1, were removed, and I believe the differences are daily increasing. Of the whole number amputated in the early period, 1 in 9 died; in the later, 1 in 3 2-5ths. Cured, including amputations, in the early period, 40, or 1 in $1\frac{3}{4}$; in the later 48, or 1 in $1\frac{3}{4}$ very nearly. Cured without amputation in the early period, 13, or 1 in 5.4; later period, 31, or 1 in 2.6. From which it appears that the number of cures in the two periods is nearly precisely the same, but that in the early period it is effected principally by the amputating knife, in the later by treatment.

Another practical conclusion appears to be, that in disease requiring amputation, after the acute stage has passed, the earlier the amputation is performed the better is the chance of recovery. This is a conclusion at which I have long since arrived, and I would lay it down as an axiom, that the shorter the duration of the disease, and the less the system has suffered under it, the greater is the chance of recovery after amputation, and *vice versa.* The difficulty is to determine, in the early stage, what disease is curable without amputation, and what must ultimately be removed; a correct diagnosis here, as in almost every other instance, being essential to success. The error we at present commit is delaying amputation after every other rational hope of cure has fled, merely because the patient is not obviously dying. The consequence has been the reduction of the success of amputations from 1 in 30 to 1 in less than three by one calculation, and from 1 in 9 to 1 in 3 2-5ths by another. To this point I would beg to call the attention of hospital surgeons in this section. Our predecessors amputated to remove what they reckoned an incurable disease, while the health was still good—we seldom operate except to save life, or rather to preserve from immediate death. Now I feel satisfied, that the number of lives rapidly ebbing from whatever cause, which amputation will save, is very small; and if we restrict our amputations to such cases, we must necessarily be very unsuccessful. I am no advocate for uncalled-for operation; but surely it is better to save life by removing a disease which we cannot cure, than to delay until treatment and amputation are nearly alike unavailing. A medium between the early and modern practice of our hospitals would preserve limbs and save lives. It is, however, but justice to ourselves to state, that the more dissipated habits and lowered condition of our patients, added to the increased size of our hospital, and the crowded state of its wards, must have considerable influence in diminishing the success of our operations.

*Primary amputations.*—Of the 276 cases, 77, or 1 in 3-5, are primary. Of these 38 were cured, 39 died, the recoveries and death being very near equal.

The following table shows the results in each limb:

| | *No. of each.* | *Cured.* | *Died.* | *Deaths to recoveries as* |
|---|---|---|---|---|
| Shoulder joint - | 3 | 1 | 1 | 2 to 1 |
| Arm - - - - | 23 | 12 | 11 | 11 to 12 |
| Forearm - - - | 15 | 15 | 0 | No death. |
| Hip - - - - | 1 | 1 | 0 | No death. |
| Thigh - - - | 12 | 1 | 11 | 11 to 1 |
| Leg - - - - | 22 | 7 | 15 | 2 to 1 fully. |
| Foot (partial) - | 2 | 2 | 0 | No death. |
| Total | 77 | 38 | 39 | |

The remarkable circumstance in these results is the great mortality. As it stands the deaths exceed the recoveries; and if we exclude the forearm, all of which recovered, the disproportion becomes very great. When we compare this table with that of Mr. Guthrie, the discrepancy is extraordinary. In his primary operations the deaths are to the recoveries as 1 to 12; in ours they rather exceed the recoveries. I believe that in military practice, especially on the field of battle, very many limbs were removed which, in civil practice, we should have attempted to preserve. In our hospital we rarely, if ever, amputate, unless it be obvious that the limb cannot possibly be saved. It is farther probable, that when the patient is seen almost immediately after being wounded, and the amputation is performed on the field of battle, he is in a much better state to bear the operation, and more favourably circumstanced for recovery, than in civil practice, where he is not seen by the hospital surgeon for many hours after the accident, and after being carried, it may be, several miles. Our accidents demanding amputation are rarely uncomplicated; a fall from a height, or the descent of a mass of earth, not only smashes a limb, but injures another limb, the head or other important organ, and inflicts a severe shock on the general system. From these causes the majority of our patients are in such a state of extreme exhaustation and shock when admitted, as to be unable to bear the operation; and when they rally the reaction is too often typhoid, or attacks a vital organ. Our machinery and railway accidents, which form a considerable proportion of our primary amputations, are also very frequently complicated, and of themselves severely implicates the constitution. To these must be added the risks our patients run after the immediate effects of the amputation are past, as explained in the sequel. Whether these considerations satisfactorily account for our great mortality it is not easy to determine.

Mr. Guthrie, in his work so frequently alluded to, insists much on the impropriety of operating while the constitution is much under the influence of shock. In this I fully concur, and I would lay it down as an axiom in primary amputations, that the less there is a shock, or in other words, the nearer the patient is to a state of health, the better will he bear the operation, and the more certain will be his recovery; and I believe it is of the effects of fearful depression that so many of our patients perish.

The second striking peculiarity in our tables is the fatality of our primary amputations of the thigh. In twelve cases there is but one recovery.

*Secondary amputations.*—Of the 276 cases, 46, or 1 in 6, were secondary. Of these there were—Cured, 20; Died, 26; Deaths to Recoveries as 5 to 4 fully.

The following table shows the detailed results:—

| | No. of each. | Cured. | Died. | Deaths to recoveries. |
|---|---|---|---|---|
| Shoulder-joint . | 1 | 1 | 0 | No death. |
| Arm . . . . | 13 | 6 | 7 | 7 to 6. |
| Forearm . . . | 3 | 3 | 0 | No death. |
| Thigh . . . | 24 | 8 | 16 | 2 to 1. |
| Leg . . . . | 5 | 2 | 3 | 3 to 2. |
| Total | 46 | 20 | 26 | 5 to 4 fully. |

This table *apparently* confirms the experience of our army surgeons, inasmuch as the *aggregate* result of secondary amputation is even more unfavourable than of primary. It, however, illustrates well the necessity of analyzing tables, because so far from proving that secondary are less favourable than primary amputations, it shows the contrary to be true. In order that inferences drawn from tables of this kind be accurate, the amputations performed *on each limb* must be compared, otherwise they are almost certain to be erroneous. The following table illustrates this:—

*Comparison of the results of primary and secondary amputations of each limb.*

| | Primary deaths to recoveries. | Secondary deaths to recoveries. |
|---|---|---|
| Shoulder-joint . . | 2 to 1. | No death. |
| Arm . . . . . . | 11 to 12. | 7 to 6. |
| Forearm . . . . | No death. | No death. |
| Thigh . . . . . | 11 to 1. | 2 to 1. |
| Leg . . . . . . | 2 to 1 fully. | 3 to 2. |

This table shows that in the thigh secondary amputations are much more favourable than primary, in the leg decidedly so, in the forearm equal, in the shoulder superior, and in the arm slightly inferior. The numbers in some instances are small, and require confirmation. The *apparently* more favourable results of primary and secondary amputations in the *aggregate* depends on the greater number of amputations of the forearm (fifteen primary, three secondary,) and on two partial amputations of the foot in the former, and none in the latter. It is remarkable that of twenty-two amputations of the forearm recorded in these tables, there is no death. It would be premature to draw from these tables any conclusion for or against primary as compared with secondary amputations. This much I may say, that the encouragement which they hold out for primary amputations in our Infirmary is not great. The subject requires farther illustrations, and I hope will not be lost sight of. To illustrate it still farther, I have noted the results of forty compound fractures and dislocations, in which attempts were made to save the limb, and on which no secondary amputation was performed.

*Table showing the results of Compound Fractures and Dislocations (not amputated.)*

| | No. of each. | Cured. | Died. | Deaths to recoveries. |
|---|---|---|---|---|
| Thigh - - | 5 | 1 | 4 | 4 to 1 |
| Leg - - - - | 21 | 15 | 6 | 2 " 5 |
| Foot and Ankle | 5 | 2 | 3 | 3 " 2 |
| Arm - - - - | 5 | 3 | 2 | 3 " 2 |
| Elbow - - - | 2 | 1 | 1 | 1 " 1 |
| Forearm - - | 2 | 1 | 1 | 1 " 1 |
| | 40 | 23 | 17 | |

From this table it appears that in attempts to cure compound fractures and dislocations the cures considerably exceed the deaths.—The last table shows that when the attempt fails secondary is more favourable than primary amputation. The combined results seem to warrant attempts to save severe cases, in preference to immediate amputation.

*Table showing the number of Amputations performed at different Ages, with the Cures and Death distinguished according to sex, and divided into Amputations for Disease—Primary, Secondary.*

| Ages from | MALES. | | | | | | FEMALES. | | | | | |
|---|---|---|---|---|---|---|---|---|---|---|---|---|
| | Disease. | | Primary. | | Secondary. | | Diseases. | | Primary. | | Secondary. | |
| | Cured. | Died. | Cured. | Died. | Cured. | Died. | Cured. | Died. | Cured. | Died. | Cured. | Died. |
| 1 to 10 | 9 | 4 | 0 | 0 | 1 | 0 | 2 | 0 | 1 | 0 | 0 | 0 |
| 10 20 | 30 | 2 | 5 | 8 | 3 | 7 | 13 | 3 | 2 | 0 | 2 | 0 |
| 20 30 | 17 | 9 | 10 | 14 | 5 | 0 | 6 | 1 | 2 | 2 | 2 | 0 |
| 30 40 | 15 | 6 | 10 | 4 | 2 | 6 | 7 | 2 | 0 | 0 | 1 | 0 |
| 40 50 | 6 | 4 | 4 | 4 | 1 | 6 | 4 | 1 | 0 | 0 | 0 | 0 |
| 50 60 | 3 | 1 | 1 | 5 | 2 | 3 | 0 | 0 | 0 | 0 | 0 | 2 |
| 60 70 | 1 | 3 | 1 | 0 | 0 | 1 | 2 | 0 | 0 | 0 | 0 | 0 |
| 70 80 | 0 | 0 | 2 | 0 | 0 | 0 | 0 | 0 | 0 | 0 | 0 | 0 |
| 80 90 | 0 | 0 | 0 | 1 | 0 | 0 | 0 | 0 | 0 | 0 | 0 | 0 |
| Totals. | 81 | 29 | 33 | 36 | 14 | 23 | 34 | 7 | 5 | 2 | 5 | 2 |
| | 110 | | 69 | | 37 | | 41 | | 7 | | 7 | |
| | 216 | | | | | | 55 | | | | | |
| | 271 | | | | | | | | | | | |

This table contains only 271 cases, because 5 of the 276 of which the former tables are composed had no age given.

The following particulars may be gathered from it :—

Of 216 males 110 were amputations for disease, and 106 (nearly one-half) for accidents. Of 55 females 41 were for disease, and 14 (about a fourth) for accidents.

Of 110 males for disease there were—Cured, 81; Died, 29; Deaths to Recoveries, as 1 to 28.

Of 41 females there were—Cured, 34; Died, 7; Deaths to Recoveries, as 1 to 4.8, exhibiting a proportion decidedly in favour of females.

Of 106 male amputations for accidents there were—Cured, 47; Died, 59; Deaths to Recoveries, as 1 11-47ths to 1.

Of 14 females for accidents there were—Cured, 10; Died, 4; Deaths to Recoveries, as 2 to 5, again giving a result in favour of the females.

*Table showing the number of amputations at different ages (including males and females) with the proportionate mortality.*

| *Ages* | | *Numbers.* | *Cured.* | *Died* | *Deaths to recoveries.* |
|---|---|---|---|---|---|
| From 1 to | 10 | 15 | 11 | 4 | 1 to 2 3-4ths. |
| 10 | 20 | 48 | 43 | 5 | 1 to 8 3-5ths. |
| 20 | 30 | 33 | 23 | 10 | 1 to 2 3-10ths. |
| 30 | 40 | 30 | 22 | 8 | 1 to 2 1-4th |
| 40 | 50 | 15 | 10 | 5 | 1 to 2 |
| 50 | 60 | 17 | 6 | 11 | 2 to 1 nearly. |
| 60 | 70 | 8 | 4 | 4 | 1 to 1 |

The rate of mortality for the ages between 9 and 20 is by much the most favourable (1 to 8 and 3-5ths,) while that from 50 to 60 is the least so (2 to 1 ;) with the exception of those from 60 to 70 (1 to 1,) all the others vary only from 1 to 2 the highest, to 1 to 2 and 3-4ths the lowest.

The ages from 10 to 20 afford the greatest number of amputations for disease; those from 20 to 40 the greatest number for accident.

*Causes of death in amputations.*—I have been able to procure pretty accurate pathological accounts of 73 deaths after amputation, all occurring in the hospital. I have arranged them as follows :—

*Table shewing the causes of death in 73 amputations, the limbs amputated, and the number of days each case survived.*

| | | *Primary.* | *Secondary.* | *Diseased.* | *Shoulder.* | *Arm.* | *Thigh.* | *Leg.* | *Total.* | *Number of days each case survived.* |
|---|---|---|---|---|---|---|---|---|---|---|
| | Bad sores | 0 | 1 | 0 | 0 | 0 | 1 | 0 | 1 | 39 |
| | Cerebral effusions | 0 | 0 | 1 | 0 | 0 | 0 | 1 | 1 | 32 |
| | Diffuse inflammation and erysipelas | 3 | 1 | 0 | 0 | 3 | 1 | 0 | 4 | 17, 7, 11, 5 |
| | Exhaustion, shock, and complications | 5 | 6 | 0 | 0 | 2 | 9 | 0 | 11 | ?, ?, 2 hrs., 3,1,12hrs.,1h. |
| | Delirium tremens | 0 | 1 | 0 | 0 | 0 | 1 | 0 | 1 | 3, 4 |
| | Gangrene of stump | 4 | 2 | 0 | 0 | 0 | 4 | 2 | 6 | 9 hrs., 3, 3, 1, 5, 7 |
| | Secondary hæmorrhage | 0 | 2 | 2 | 0 | 0 | 4 | 0 | 4 | 49, 2, 2, 2 |
| | Tetanus | 1 | 0 | 0 | 0 | 0 | 1 | 0 | 1 | 1 |
| Secondary Inflam. 44. | Diarrhœa | 2 | 1 | 2 | 0 | 1 | 3 | 1 | 5 | 5, 8, 20, 3, 11 |
| | Pericarditis (29) | 1 | 0 | 1 | 0 | 1 | 0 | 1 | 2 | ?, 21 |
| | Pleuritis (29) | 1 | 4 | 1 | 0 | 1 | 4 | 1 | 6 | ?, 94, 4, 6, 12, ? |
| | Pneumonia (29) | 2 | 0 | 0 | 0 | 0 | 1 | 1 | 2 | 20, 38 |
| | Purulent deposits (29) | 4 | 5 | 7 | 1 | 5 | 3 | 7 | 16 | 13, 12, 7, 27, 24, 29, 28, 6 |
| | Phlebitis (29) | 2 | 0 | 1 | 0 | 0 | 1 | 2 | 3 | 14, 5, 17, 18, 18, 5, 10, 36 |
| | Rigors | 5 | 1 | 1 | 1 | 3 | 1 | 2 | 7 | ?, 14, 18, 3, 12, 45, 22 |
| | Secondary inflammation | 2 | 0 | 0 | 0 | 0 | 1 | 1 | 2 | 10, 6 |
| | Secondary external abscess | 0 | 0 | 1 | 0 | 0 | 0 | 1 | 1 | 31 |
| | | 32 | 24 | 17 | 2 | 16 | 35 | 20 | 73 | |

From this table it appears that of 73 deaths, of whose causes and pathology more or less accurate records are given, 32 were primary, 24 secondary, and 17 disease. I have divided the causes of death into, 1st, causes independent of secondary inflammation; and 2d, the various kinds of secondary inflammatory affections.

Of the 8 causes enumerated belonging to the first class, 4 caused one death each, and need not detain us; of the other four, exhaustion, shock, and complication of injuries, caused 11, gangrene of the stumps, 6, secondary hæmorrhage 4, and diffusive inflammation and erysipelas 4. It is remarkable that of these 25 deaths 22 were from accidents, and 3 from disease—of which three, two were caused by secondary hæmorrhage. It is highly probable that the 16 cases of exhaustion and gangrene mainly owed their fatal termination to the great severity of the accidents which rendered amputation necessary, aided by the severity of the operation.—This is confirmed, first, by the number of hours and days which each case survived the amputation (four did not survive one day, and the longest period is seven days;) and, second, by the part amputated (of the sixteen, fourteen were thigh, two leg.) The diffusive inflammation and erysipelas were probably owing to the severity of the injury, and the air of the hospital. I was surprised to find that so few of the fatal terminations are attributable to these causes: of the four, three were arm, one thigh.

*Secondary inflammations.*—In this class, besides those pathological states which are almost universally acknowledged to arise from what is usually styled secondary inflammation, I have included "rlgours" and "diarrhœa," the former because I have no doubt that the rigours were symptomatic of secondary disease, and the latter because I believed that ulceration of the intestinal mucous membrane causing diarrhœa belongs to the same class of affections. Of the seventy-three deaths, forty-four belong to secondary inflammation, and twenty-nine to other causes, showing that secondary disease exceeds all other causes of death combined, very nearly in the proportion of five to three. Of these forty-four, nineteen were primary, eleven secondary, and fourteen diseased.

As this subject, secondary inflammation, is one of vast importance, I may, perhaps, be excused if I state what appear to me a few facts connected with it. First, as regards their history, I believe they are much more common now than they were thirty or forty years ago. My reason for saying so is, that amputations were then much more successful than they are now, which could not have been the case had these fatal secondary inflammatory diseases been as frequent. If I were asked to assign the probable cause, I should say that it is because those amputations were performed at a much earlier stage of disease, and on patients comparatively in good general health; second, they are infinitely more common in hospitals than private practice. A large proportion of our hospital surgical deaths arises from them, while in private they are rare. The reason here is obvious—the air of the hospital debilitating the constitution. Third, the more severe the person's disease or injury, and the more the system has suffered, the greater is the liability to these affections. This is almost a corollary on the fact just stated. Fourth, the more severe the operation, the more liable is the patient to secondary inflammation. Of the forty-four cases, fourteen were thigh, seventeen leg, two shoulder-joints, and eleven arm. Not one of the amputations of the forearm was followed by secondary disease. I am well aware that these secondary inflammations occasionally follow very trivial operations and injuries. I have lost two cases of fistula in ano from this cause, and more than one injury of the feet and hands, but these were all in hospital, and were probably attributable to the atmosphere. Fifth, there is a strong tendency in surgical disease to localize itself in some important internal organ, especially the lungs; and this is most apt to occur in the feeble and exhausted—in many cases I believe a very short time before death. I believe that a careful examination would show that the majority of those patients who die of discharge and exhaustion exhibit these affections, and that they are most liable to occur when the discharge is most profuse. I am well aware that profuse discharge,* or even an open sore,† is by no means necessary to their formation; but I feel satisfied that a statistical investigation would show that a great majority occur during the continuance of profuse unchecked suppuration. In cases where there was no discharge, and little, if any pus, secreted, I have remarked the presence of lobulated pneumonia without deposit of pus.

I have introduced the above remarks principally with the view of making them bear on what appears to me a retrograde movement in the treatment of stumps of late years becoming fashionable—union by the second intention—a proposal sanctioned by the hope of preventing secondary inflammation after amputations. If the above statements are correct, this method of treatment rests on very false principles, and cannot be too soon reconsigned to that oblivion in which, in Great Britain, it slumbered for so many years. I believe in all cases union by the first intention, by avoiding the risks of a suppurating stump, diminishes "pro tanto" the dangers of an attack of secondary disease.

To this view it is objected that many patients who suffer amputation for disease profusely suppurating, die within a few days of the performance of the operation, apparently in consequence of the sudden check given to the discharge. In a great many of these cases I am convinced that the secondary inflammation existed before the operation, and that it was the failure of strength, caused by these latent inflammations, which induced the surgeon to perform an operation which their presence rendered altogether futile. I do not think we are yet sufficiently alive to the impropriety of amputating after the occurrence of rigors. Almost as certainly as we do amputate our patient dies. We are still less alive to the impropriety of amputating after some symptom previously mild has become suddenly aggravated. It is too much the practice to wait for some occurrence so urgent as to *demand* removal of the limb. Now the very urgency of the symptom, especially if of sudden accession, is often the strongest of all reasons for not amputating, as it too frequently depends on secondary disease, which will certainly destroy our patient whether we amputate or not.—*Lond. Med. Gaz.*

* Case of fistula in ano nearly healed.

† Paterson—abscess of prostate, 18th of July, 1834.

